# Trends in depressive symptoms in Germany’s adult population 2008–2023

**DOI:** 10.1007/s00127-025-02965-6

**Published:** 2025-07-22

**Authors:** Lena Walther, Stephan Junker, Petra Rattay, Ronny Kuhnert, Heike Hölling, Elvira Mauz

**Affiliations:** 1 Department of Epidemiology and Health Monitoring, Robert Koch Institute, Berlin, Germany; 2 Institute of Medical Sociology and Rehabilitation Science in Berlin, Charité - Universitätsmedizin, Berlin, Germany

**Keywords:** Depression, Trends, Germany, Survey data, Adults, Mental health surveillance

## Abstract

**Purpose:**

The present study examines trends in the prevalence of depressive symptoms among adults in Germany between 2008 and 2023 within the framework of mental health surveillance.

**Methods:**

We used survey data from seven observation periods from a total of *N* = 74,096 randomly sampled participants aged 18–79 living in Germany. Symptoms of depression were measured using the Patient Health Questionnaire-8. Time series consisting of prevalence estimates were calculated, also for subgroups by sex, age and education. Developments in the more severe symptom range and in individual symptoms were also examined. Joinpoint regression and logistic regressions including linear splines were used to assess trends.

**Results:**

Depressive symptom prevalence rose 2008–2023, with stagnation 2008–2020 followed by an increasing trend 2020–2023. While 7.5% of adults were at or above screening cutoff in 2020, 14.8% were affected in 2023. This development was found across subgroups. Both moderate and more severe symptoms increased, with a particular rise in severe symptoms among males. A clearly increasing trajectory was found across symptoms, although developments in low self-worth/guilt were less clear. Depressive symptoms were generally more prevalent among females than males, the youngest than the eldest adults and those with lower educational attainment. Young females and males with low educational attainment were particularly affected in the final observation period.

**Conclusions:**

The observed increases in depressive symptoms among adults in Germany in times of multiple collective crises call for efforts to promote and protect mental health as well as for continued observation.

**Supplementary Information:**

The online version contains supplementary material available at 10.1007/s00127-025-02965-6.

## Introduction

Depressive disorders such as major depressive disorder (MDD) are constituted by symptoms including low mood, anhedonia, fatigue or low energy, feelings of worthlessness or guilt, sleep disturbances, changes in appetite, concentration problems, psychomotor retardation or agitation and thoughts of death or suicidality [1]. They are highly prevalent and considered to be among the biggest contributors to the burden of disease globally, with wide-ranging negative consequences for individuals and societies [2, 3]. Depression is often comorbid with other mental disorders, particularly anxiety disorders [1], as well as somatic disorders [3], and represents a major risk factor for suicide [4]. Females are generally more likely to experience depression than males [1, 5–9], although gender-specific clinical manifestations may result in an underestimation of prevalence among males [10]. The relationship between depression risk and age is less clear [1, 6, 9]. There is evidence that younger adults are more likely to develop major depression and that dysthymia is especially common among middle-aged adults, while older age comes with unique depression risk factors [1] and possibly unique clinical expression [11]. Different patterns by age have also been found in studies contrasting results of symptom screening, psycho-diagnostic interviews and self-reports on clinician diagnosis [6, 9]. Low socioeconomic status [12] and low level of education specifically [9, 13, 14] have consistently been found to be risk factors for depressive symptoms.

Over the past decades, it has been repeatedly hypothesised that depressive disorders are on the rise in western countries owing to various facets of modern life (e.g. increasing social inequality, loneliness, chronic diseases) [15, 16]. However, empirical evidence has been mixed [17]. For example, one meta-analysis of studies with observation periods between 1976 and 2016 indeed reports a rise in depression prevalence [18], while another covering observation periods between 1990 and 2010 finds increases in psychological distress but not in depression rates when taking population growth and age structure into account [19].

More recently, the COVID-19 pandemic was widely assumed to have had negative impacts on the mental health of populations worldwide [20]. Again, international evidence has been mixed, with reviews highlighting a large heterogeneity in findings [21]. However, several reviews concluded that mental health did seem to have declined in the early stages of the pandemic [21–23]. There is far less evidence on later stages or post-pandemic developments in a period characterised by several other crises, including wars with global impacts, inflation and cost-of-living crises in many countries, and a worsening climate crisis. Data from France [24], Belgium [25] and the United States [26] shows similar or lower depressive symptom levels in 2022/2023 as/than in 2020/2021. However, Belgian survey data shows a lasting increase in depressive symptoms 2020 to 2022 [25] compared to 2001 to 2018 [27]. Similarly, a Dutch nationally representative psycho-diagnostic interview study showed a near doubling of mood disorder prevalence from 6.0 to 10.8% between 2007–2009 and 2019–2022 with no difference between the pre- and peri-pandemic part of this latter time period [28]. A psycho-diagnostic interview study in Canada revealed an increase in the 12-month prevalence of major depression from 4.7% in 2012 to 7.6% in 2022 [29].

With regard to developments in Germany, routine health care system data showed a rise in the administrative prevalence of depression. A depression diagnosis (ICD codes F32, F33, F34.1) was documented for 13.9% of those who had any contact with a health care provider in an outpatient setting in 2022 compared to 12.1% in 2012 according to nationwide claims data on all statutorily insured individuals [30]. Multiple health insurance providers have also reported a rise in mental health-related and depression-related sick leave with data from until 2023 [31–34].

Survey data covering longer time spans is scarce. Data from Germany’s Socioeconomic Panel showed an overall trend of improvements in a mental health-related quality of life measure that has been found to be associated with symptoms of depression [35, 36] between 2002 and 2020 [37], with dips in 2010 after the financial crisis and in the first year of the COVID-19 pandemic. Data from the same panel also showed increases in depressive symptoms between 2019 and peri-pandemic 2020 as well as 2021 [38]. A large cohort study in Germany similarly found a symptom increase in peri-pandemic 2020 compared to baseline (2014–2019) [39]. However, most German studies focused on the early stages of the pandemic with few reports on more recent developments [40, 41] and none taking a longer-term view.

Overall, long-term, systematic surveillance of trends in depression and depressive symptoms as well as other mental disorders and their symptoms has been lacking in Germany [42]. The assessment of developments over time as well as of symptom burden distribution between population subgroups within dedicated public health surveillance systems represents an important evidentiary basis for public health practice [43, 44]. To this end, mental health surveillance has been in development at the Robert Koch Institute since 2019 [42]. In response to new information needs arising in the COVID-19 pandemic, one of the first activities within mental health surveillance was the development of a high-frequency surveillance approach based on monthly estimates for just a few indicators [45]. Contrary to the findings outlined above, the observation of two core symptoms of depression between 2019 and 2023 actually showed initial declines in symptoms at the start of the pandemic [46] followed by substantial increases between 2020 and spring 2023 alongside increases in two symptoms of anxiety and declines in self-rated mental health between 2021 and 2023 [46, 47]. Recent developments in depressive symptoms have not been investigated using a broader screening instrument. They have also not been examined in the context longer time series including data from earlier Robert Koch Institute health monitoring studies, which captured symptoms of depression in 2008–2011 [5], 2014–2015 [48] and 2019–2020 [49].

The present study analyses trends in eight depressive symptoms in Germany’s adult population over a time period of 15 years. To this end, more recent data from 2021 to 2023 is placed within the context of longer time series with data from 2008 to 2020. We calculated standardised estimates of the prevalence of depressive symptoms, examining trajectories of both moderate and more severe symptom levels as well as individual symptoms. To examine developments in the risk of depressive symptoms by population subgroup, we compared trends between groups by sex, age and level of education.

## Methods

### Data

Data used in this study was collected in six different population-based surveys of adults living in Germany conducted within the framework of health monitoring at Germany’s public health institute, the Robert Koch Institute: the German Health Interview and Examination Survey for Adults (DEGS1, 2008–2011 [50, 51]), German Health Update (GEDA)-European Health Interview Survey (EHIS) 14/15 [52, 53], GEDA-EHIS 19/20 [54], GEDA 21, GEDA 22 and GEDA 23. DEGS1, GEDA-EHIS 14/15 and the subsequent GEDA surveys differ in sampling design and survey mode. DEGS1 and GEDA14/15 encompassed samples randomly drawn from records of the residents’ registration office using a two-stage cluster sampling approach. Approximately half of the sample in DEGS1 comprised re-interviewed participants from the study BGS98 [50, 55]. DEGS1 used paper and pencil interviews conducted in a study centre. GEDA-EHIS 14/15 used a mixed-mode design of online interviews (44,6%) and paper pencil interviews (55,4%) completed by participants at home. GEDA-EHIS 19/20, GEDA 21, GEDA 22 and GEDA 23 data comes from telephone interviews with individuals sampled in a dual-frame procedure randomly drawing from mobile and landline telephone numbers. All interviews were conducted in German. Response rates were 64% for re-interviewed participants in DEGS1 and 42% for newly recruited DEGS1 participants; 26,9% for GEDA-EHIS 14/15; 21,6% for GEDA-EHIS 19/20; between 17.6% and 22.5% per month for GEDA21 (monthly recruitment); 16.1–19.8% per month for GEDA 22; and 17.4–18.9% for GEDA 23. We used each survey as a whole with the exception of GEDA-EHIS 19/20, which we separated into a pre-pandemic observation period and a peri-pandemic observation period (cut point: March 16, when containment measures began in Germany).

**Table 1:** Sample characteristics

	DEGS1	GEDA-EHIS 14/15	GEDA-EHIS 19/20 a)	GEDA-EHIS 20 b)	GEDA 21	GEDA 22	GEDA 23
*Data collection* *period*	11/2008 to 2/2012	11/2014 to 7/2015	4/2019 to 3/2020	3/2020 to 1/2021	6/2021 to 12/2021	2/2022 to 1/2023	1/2023 to 2/2024
N Total	7,987	22,909	12,843	8,056	4,479	7,352	10,470
Female	3,789 (49.7%)	10,321 (49.3%)	6,220 (50.7%)	3,740 (48.1%)	2,166 (50.0%)	3,376 (49.9%)	4,954 (49.9%)
Male	4,198 (50.3%)	12,588 (50.7%)	6,623 (49.3%)	4,316 (51.9%)	2,313 (50.0%)	3,976 (50.1%)	5,516 (50.1%)
Age 18–29	1,072 (18.7%)	3,888 (17.8%)	1,393 (18.7%)	708 (15.6%)	406 (17.5%)	615 (17.5%)	964 (17.5%)
Age 30–44	1,730 (25.2%)	5,325 (23.3%)	2,441 (26.1%)	1,328 (21.8%)	795 (24.9%)	1,304 (25.0%)	1,753 (24.4%)
Age 45–64	3,136 (36.5%)	8,977 (38.3%)	5,467 (37.2%)	3,514 (39.9%)	1,957 (38.2%)	3,130 (38.0%)	4,361 (38.3%)
Age 65–79	2,049 (19.6%)	4,719 (20.6%)	3,542 (18.0%)	2,506 (22.7%)	1,321 (19.5%)	2,303 (19.5%)	3,392 (19.9%)
Low level of education	2,551 (36.6%)	4,978 (29.5%)	2,170 (26.4%)	1,361 (26.9%)	632 (24.5%)	1,060 (24.3%)	1,344 (22.4%)
Medium level of education	3,844 (47.6%)	12,480 (54.4%)	5,912 (55.1%)	3,542 (53.0%)	2,037 (55.4%)	3,270 (55.1%)	4,786 (57.3%)
High level of education	1,502 (14.5%)	5,405 (15.8%)	4,729 (18.0%)	3,130 (19.6%)	1,793 (19.5%)	3,002 (20.1%)	4,311 (19.7%)
Missings on education	90	46	32	23	17	20	29
Missings on PHQ-8	447	354	205	162	85	253	282

*Number of observations unweighted; percentage weighted as well as sex- and age-standardised. GEDA-EHIS 19/20 (a) covers a pre-COVID-19 pandemic period (4/19 − 3/20)*,* GEDA-EHIS 20 (b) covers a peri-pandemic period (3/20 − 1/21).*

Across surveys, women tended to be more likely to participate than men, older adults were more likely to participate than younger adults, and the likelihood of participation increased with level of education. Because DEGS1 included adults aged 18 to 79, we limited the samples of all surveys to this age range for the present study, resulting in a total sample of *N* = 74,096. See Table 1 for an overview of sample characteristics.

### Weighting and standardisation

Design and adjustment weighting procedures were used to produce representative estimates for the adult population. Design weighting accounted for different selection probabilities for all surveys and stratification in sampling for DEGS1 and GEDA-EHIS 14/15. For DEGS1 and GEDA-EHIS 14/15 design weighting also accounted for clustered sampling (see details in [50, 53]). For the telephone surveys (GEDA-EHIS 19/20 onward), design weighting accounted for the dual-frame sampling procedure (see details in [54, 56]). Adjustment weighting was by sex, age, level of education (ISCED) and region (as well as nationality for DEGS1). Information on the distribution of these features in the population at the times of the respective surveys came from the Federal Statistical Office (Destatis) and the German Microcensus.

The changing age structure of the population was taken into account by means of sex and age standardisation using the respective distributions of the European standard population from 2013 [57]. For comparisons between education groups over time, sex and age standardisation was also applied within each level of education group such that the sex and age distributions between groups were matched, while the proportion of each level of education was left intact.

### Measures

Symptoms of depression were measured using the self-report eight-item version of the Patient Health Questionnaire’s depression module (PHQ-8 [58]). Originally developed to screen for major depressive disorder in primary care settings, the PHQ-8 has become a widely used tool for assessing current depressive symptoms and their severity in clinical as well as population-based studies. With the exception of suicidality, the scale’s eight items capture all diagnostic criteria of major depression listed in the Diagnostic and Statistical Manual of Mental Disorders, 4th edition (DSM-IV): depressed mood, anhedonia, sleep disturbances, fatigue, changes in appetite, low self-worth or guilt, concentration problems, psychomotor retardation or agitation. Respondents are asked to report the frequency with which they have felt bothered by each of these symptoms over the last two weeks (0 = “not at all”, 1 = “several days”, 2 = “more than half the days”, 3 = “nearly every day”), resulting in total scores ranging from 0 to 24. A screening cutoff score of 10 is recommended [58] and was confirmed as the cutoff maximizing combined sensitivity (0.86; 95% CI 0.80 to 0.90) and specificity (0.86; 95% CI 0.83 to 0.89) for detecting MDD when compared to semi-structured diagnostic interviews in a more recent meta-analysis based on individual participant data [59]. The PHQ-8 can also serve as a dimensional measure using the raw score or the following five severity levels [58, 60]: none (0–4), mild (5–9), moderate (10–14), moderately severe (15–19), severe (> 20). In the present study, we report the proportion of the population with potentially clinically significant symptoms of depression (PHQ-8 ≥ 10) as well as the proportion with moderately severe or severe symptoms (PHQ-8 ≥ 15). We also look at developments in each of the eight individual symptoms of depression by estimating the proportion who reported experiencing each symptom “more than half the days” or “nearly every day” in the previous two weeks.

Results are stratified by sex, age and level of education. Self-reported sex assigned at birth was used to stratify into female and male because information on gender identity was not ascertained in the 2008–2011 or 2014–2015 surveys. Age was grouped as follows: 18–29, 30–44, 45–64 and 65–79 years. Educational attainment was used as an indicator of socioeconomic status. Three categories of low, medium and high level of education were formed according to the CASMIN classification [61] based on self-reported school and vocational qualifications.

### Statistical analyses

We calculated weighted and unstandardised as well as weighted and sex- and age-standardised estimates for the percentage of PHQ-8 ≥ 10, also stratified by sex, age, level of education, by age and sex and by level of education and sex, along with 95% confidence intervals. We also calculated estimates for the percentage of PHQ-8 ≥ 15 and for the prevalence of individual symptoms for the total sample and stratified by sex.

Based on the plotted estimates and previous findings showing increases in the prevalence of the first two PHQ-8 symptoms from late 2020 onward [46, 47], we hypothesised a change in trend at the 2019/2020 survey. We used joinpoint analysis [62] to assess the presence of change points in the overall trend. We used the default parameters, meaning that for our time series comprising seven estimates, a maximum of one joinpoint would be identified. The weighted Bayesian information criterion (WBIC) served as selection criterion. We assumed autocorrelation between standard errors and estimated the correlation coefficients from the data.

Following the identification of a change point, we used logistic regression models including linear splines [63] for time in years to assess trends before and after the change point [64, 65], placing the knot at the position of the detected change point. We also compared the slopes of the two splines and the slopes of the splines for the time period after the change point between subgroups using interactions of subgroups and splines, see the model specification in pseudo code below.

$$\:y=lsp\:line\left(years\right)*sub\:group$$

We did not examine interactions between subgroups and the spline for the time period before the change point because (a) more recent developments in mental health vulnerabilities are more relevant and (b) design differences between the early surveys render developments more difficult to interpret. We applied a significance level of α = 0.05 to test for differences in slope.

Each of the surveys included in the analyses was treated as a single time point. For the purposes of representing time between surveys, we determined the temporal midpoint of each survey’s field time. For analyses that required continuous integer indicators (joinpoint regression), we used a time variable with yearly intervals rounding up or down from the calculated midpoint day. A robustness check of the analysis using exact days on a scale of days for the population estimates yielded similar results. For analyses that allowed for rational numbers (logistic regression with splines), we used exact dates but still on a scale of years.

The clustered structure of DEGS1 and GEDA14/15 was taken into account in all analyses. Observations with any missing responses on the PHQ-8 or on education attainment were deleted case-wise (see missings in Table 1). Analyses were conducted with R version 4.3 [66]. Joinpoint 5.0.2 was used for the joinpoint analysis [62].

## Results

**Fig. 1:**
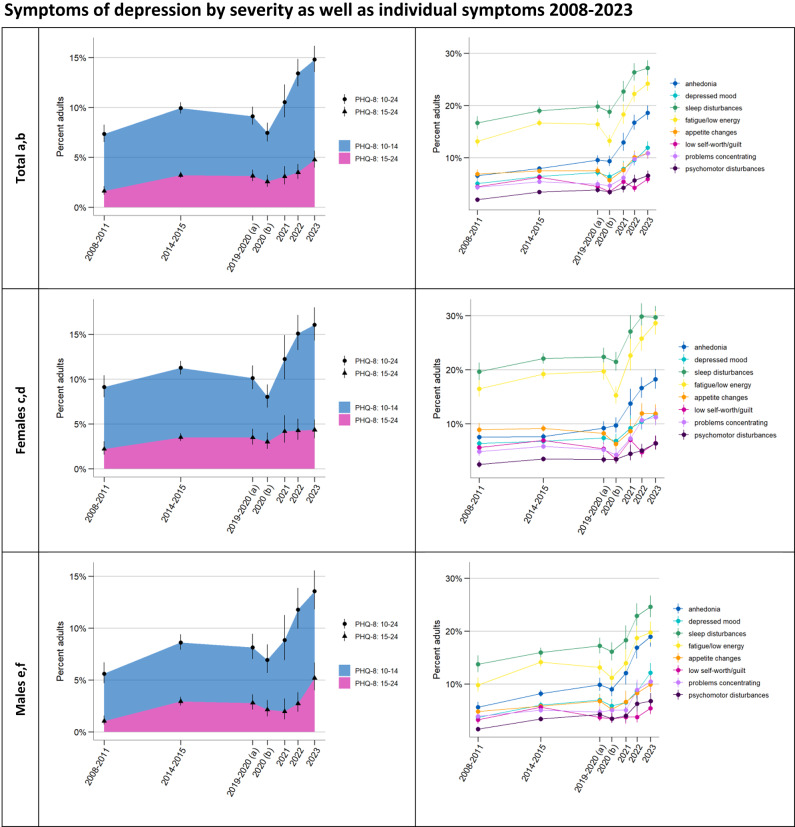
**a** Weighted and sex- and age-standardised percentage of adults (18–79 years) with symptoms of depression (PHQ-8 ≥ 10, circles) and with moderately severe or severe symptoms (PHQ-8 ≥ 15, triangles) as well as proportion of moderate symptoms (PHQ-8 = 10–14) versus moderately severe or severe symptoms (PHQ-8 = 15–24) shown as blue and pink areas. **c** Same as a for females, age-standardised and **e** males, age-standardised. **b** Weighted and sex- and age-standardised percentage of adults (18–79 years) reporting a frequency of “more than half the days” or “nearly every day” in the previous two weeks for each of the eight symptoms captured by the PHQ-8. **d** Same as b for females, age-standardised and **f** males, age-standardised. For the purposes of representing time between surveys, we plotted each estimate at the temporal midpoint of the respective survey’s field time. 2019–2020 (a) covers a pre-COVID-19 pandemic period (4/19 − 3/20), 2020 (b) covers a peri-pandemic period (3/20 − 1/21). There are survey design differences between the 2008–2011, the 2014–2015 survey and the 2019–2023 surveys (constant methodology 2019–2023) (see Methods)

Figure 1a shows sex- and age-standardised estimates for the percentage of adults with potentially clinically significant depressive symptoms (PHQ-8 ≥ 10) between 2008 and 2023 (see Supplementary Table 1 for unstandardised estimates). An initial increase between the 2008–2011 survey (7.4% [95% CI: 6.5%; 8.3%]) and the 2014–2015 (9.9% [9.4%; 10.5%]) and pre-pandemic 2019–2020 surveys (9.1% [8.3%; 10.1%]) is followed by a decline back to 2008–2011 levels by the peri-pandemic 2020 survey (7.5% [6.6%; 8.5%]) (standardised estimates). The estimates between 2020 and 2023 then show an increasing trend with estimates for 2022 (13.4% [12.1%; 14.9%]) and 2023 (14.8% [13.6; 16.2%]) far surpassing all previous estimates. Joinpoint regression confirmed this overall trajectory, with the best fit or lowest WBIC model suggesting a change in trend from timepoint 2020 b) (WBIC: 2.82). The logistic regression with linear splines for the time period before and after the change point shows a significant difference between these two trends (*p* < 0.001) and confirms a non-zero increasing slope for 2020 to 2023 (*p* < 0.001, see Supplementary Table 2). A robustness check confirmed that this was not simply due to particularly low symptom levels in 2020, with the same finding of a non-zero increasing slope for pre-pandemic 2019/20 to 2023 (*p* < 0.001).

We find this same development for both moderate symptoms (PHQ-8 = 10–14) and moderately severe or severe symptoms (PHQ-8 ≥ 15), for which standardised estimates rose from 1.6% [1.2%; 2.1%] in 2008–2011 to 3.1% [2.6%; 3.8%] 2019–2020 (pre-pandemic) to 4.8% [4.0%; 5.7%] in 2023 (Fig. 1a and Supplementary Tables 3,4: *p* < 0.001 for non-zero slopes 2020–2023; *p* < 0.001 and *p* = 0.011, respectively, for differences in slope 2020–2023 and 2008–2020). Of those with symptoms, the proportion with moderate and the proportion with moderately severe or severe symptoms remained the same 2020–2023 (*p* = 0.122, Supplementary Table 5).

As shown in Fig. 1c and e and Supplementary Tables 2–5, all of these trends in depressive symptoms are also found among both females and males, with non-zero increasing slopes 2020–2023 for both PHQ-8 ≥ 10 and PHQ-8 ≥ 15 that are significantly different from slopes 2008–2020 among males and females. One notable difference between the sexes is that among males, the percentage with more severe symptoms of depression (PHQ-8 ≥ 15) doubled between 2022 and 2023 from 2.7% [1.9%; 3.8%] to 5.2% [4.0%; 6.7%] in a development that stands out in the time series, while there was no change in this time among females. Accordingly, the regression comparing trends 2020–2023 for PHQ-8 ≥ 15 versus PHQ-8 = 10–14 in females compared to males shows a difference between the sexes (*p* = 0.049, Supplementary Table 5): An increase in both moderate and more severe symptoms in men means that the proportion of those with more severe symptoms (PHQ-8 ≥ 15) remained the same within the group above the cutoff of PHQ-8 ≥ 10. By contrast, this proportion decreased compared to moderate symptoms in females. Females generally show a higher prevalence of depressive symptoms than males (see Supplementary Fig. 1).

Looking at the eight individual symptoms of depression captured by the PHQ-8 for the total population (Fig. 1b) and stratified by sex (Figs. 1d and f), all symptoms appear to follow the same overall trajectory of increasing prevalence, with only feelings of low self-worth or guilt (“feeling bad about yourself– or that you are a failure or have let yourself or your family down”) showing a less clear development. Across all observation periods, sleep disturbances and fatigue/low energy have the highest prevalence. Anhedonia clearly surpassed changes in appetite and became the third most common symptom among adults from 2019/2020. Time series for individual symptoms stratified by age and education are included as Supplementary Figs. 2a-d and 3a-c and show similar developments across subgroups. Low self-worth or guilt appears to have increased notably between 2022 and 2023 in the low level of education group.

**Fig.2:**
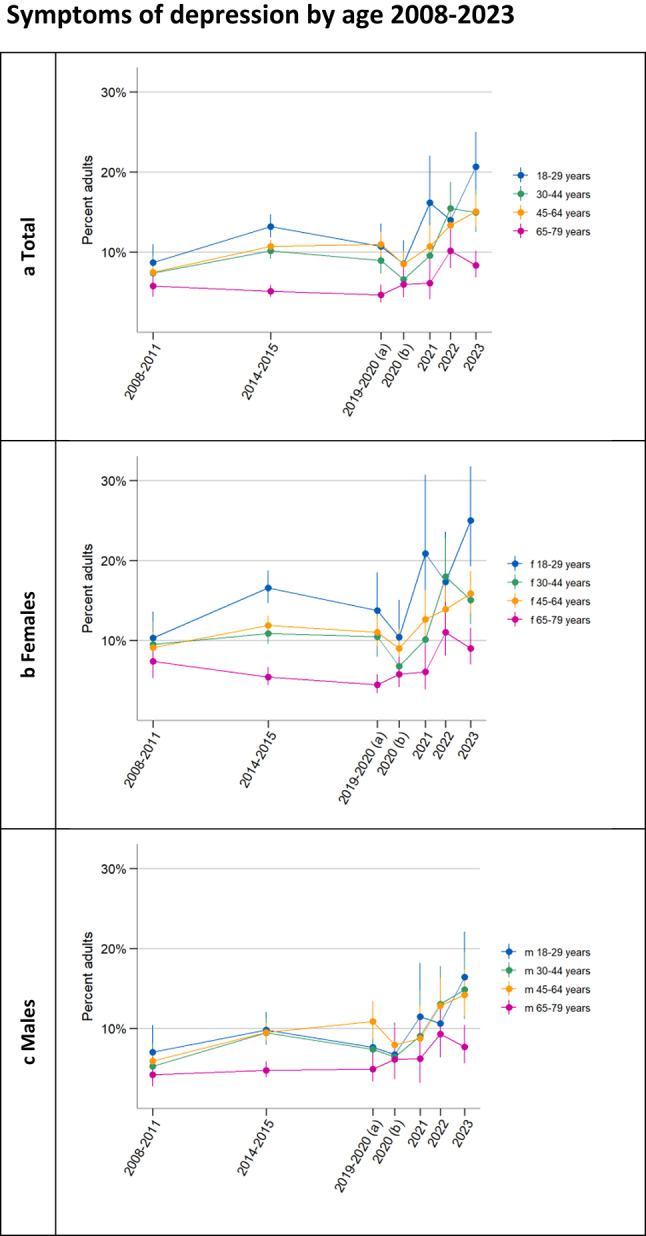
**a** Weighted and sex-standardised percentage of each age group with symptoms of depression (PHQ-8 ≥ 10) as well as weighted estimates of the percentage of **b** each age group among females and **c** each age group among males with symptoms of depression. For the purposes of representing time between surveys, we plotted each estimate at the temporal midpoint of the respective survey’s field time. 2019–2020 (a) covers a pre-COVID-19 pandemic period (4/19 − 3/20), 2020 (b) covers a peri-pandemic period (3/20 − 1/21). There are survey design differences between the 2008–2011, the 2014–2015 survey and the 2019–2023 surveys (constant methodology 2019–2023) (see Methods)

Figure 2 as well as regression results (Supplementary Table 6) show an increase in symptoms of depression between 2020 and 2023 across age groups, also stratified by sex. The youngest adults generally appeared to be at a higher risk of symptoms of depression than the eldest adults, especially among females. Young females exhibited a particularly high standardised prevalence of depressive symptoms in the final observation period (25.0% [19.2%; 31.8%]).

**Fig. 3:**
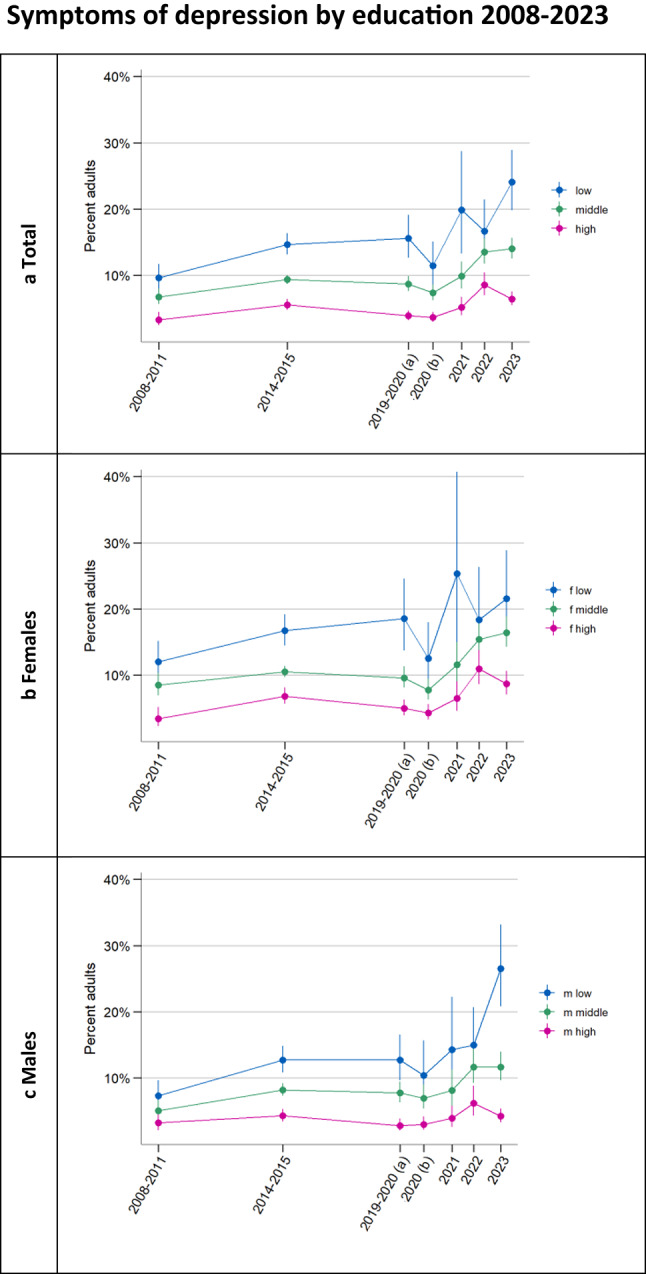
**a** Weighted and age- and sex-standardised percentage of each education group with symptoms of depression (PHQ-8 ≥ 10) as well as weighted estimates of the percentage of ***b*** each education group among females and** c** each education group among males with symptoms of depression. For the purposes of representing time between surveys, we plotted each estimate at the temporal midpoint of the respective survey’s field time. 2019–2020 (a) covers a pre-COVID-19 pandemic period (4/19 − 3/20), 2020 (b) covers a peri-pandemic period (3/20 − 1/21). There are survey design differences between the 2008–2011, the 2014–2015 survey and the 2019–2023 surveys (constant methodology 2019–2023) (see Methods)

Figure 3 also suggests potential symptom increases across education groups. Males in the low level of education group experienced a particularly striking increase in symptoms in the final observation period, with 26.5% [20.8%; 33.2%] above cutoff in 2023 compared to 15.0% [10.6%; 20.7%] the previous year and 10.4% [6.8%; 15.7%] (standardised values) in 2020. However, regression results do not confirm a trend difference in 2020–2023 between males with low and medium (*p* = 0.153) or high level of education (*p* = 0.079) and generally show increases in symptoms across education as well as sex by education groups 2020–2023 (Supplementary Table 7).

## Discussion

The present study investigates trends in symptoms of depression between 2008 and 2023 among adults in Germany by analysing survey data collected in seven different observation periods. We find that while the prevalence of potentially clinically significant depressive symptoms (PHQ-8 ≥ 10) was relatively stable with some fluctuation between 2008 and 2020, the years 2020 to 2023 saw a substantial symptom increase. This development was seen in both the moderate and the moderately severe to severe symptom range. The fact that frequency increased across almost all symptoms (trajectory less clear for low self-worth/guilt) represents further evidence for a substantive change in population mental health and confirmation of previous reports of negative developments based on just the two core depressive symptoms as well as two symptoms of anxiety and self-rated mental health [46, 47, 67]. In line with these previous findings, the results of the present study also suggest negative developments across subgroups by sex, age and level of education.

While the interpretation of developments between 2008 and 2019 is limited by changes in survey design, the observed increase in symptoms between 2019 and 2023 cannot be attributed to methodological factors because survey methods remained consistent in this time. It can be argued that the evidence for an overall rise in depressive symptoms across the observation periods is strengthened by the fact that an increase was found despite the switch from self-administered paper and pencil or online surveys 2008–2015 to an interviewer-based telephone survey from 2019 onward. The latter survey mode has been found to be associated with increased effects of social desirability and better mental health self-reports (e.g [68]). Returning to the hypothesis of a trend of rising depression prevalence in western societies [15, 16], the present findings cannot confirm a continuous increase, but do suggest an overall increase between 2008 and 2023.

To our knowledge, this is the only study showing developments in symptoms of depression in the population in Germany longer-term and beyond the earlier stages of the COVID-19 pandemic. Looking at the time period 2008–2020, there are two differences between our findings and other studies. First, the temporary increase in symptoms of depression we find between the 2008–2011 and 2014–2015 surveys contradicts findings of an improvement in mental health-related quality of life in 2014 compared to 2010 in Germany’s Socioeconomic Panel [37]. The study’s authors link poorer health in 2010 to the financial crash in 2008. This difference in findings may be due to the survey design changes in our study (self-administered paper and pencil questionnaire in study centre 2008–2011 versus paper and pencil or online at home 2014–2015) as well as the different mental health measures used. Contradictions between other studies’ finding of a symptom increase in the early pandemic [38, 39] and our finding of a symptom reduction between pre- and peri-pandemic 2019–2020 [46, 49], have been discussed elsewhere [46]. Improvements in mental health in this time may have been due to a deceleration in life [49, 69]. The prevalence of positive PHQ-8 screens found up to 2020 in our study and another large-scale, but not representative German study using the PHQ-9 are generally of the same magnitude (7.8% 2014–2019 [9] and 9.5% in 2020 [39]). To our knowledge, other population PHQ estimates for 2022 and 2023 are not available.

Except among adults aged 18–29 [30, 70], developments in outpatient depression diagnoses as documented in insurance claims data generally did not parallel the increase seen in depressive symptoms in the population between 2020 and 2023. Diagnoses increased between 2012 and 2022 but stagnated between 2020 and 2022 [30]. Various factors could have contributed to this discrepancy, including delays in treatment seeking after symptom onset and possible changes in health care use during the pandemic– both of which may differ by age. Importantly, 2022 was the first year when symptom levels surpassed all pre-2021 estimates in our survey data, meaning that possible resulting changes in diagnostic prevalence may have occurred later. Indeed, 2023 again saw a rise in outpatient depression diagnoses [70], albeit not one that stands out in the time series. Of course, it is unclear whether this recent increase or the more striking incline among 18-29-year-olds across recent years (ibid.) is due to the observed symptom increase or a result of changes in treatment seeking or diagnostic practice. By using different data sources and indicators, long-term mental health surveillance can help shed light on how developments in morbidity in the population and in diagnoses in the health care system might relate [45].

The present study does not provide evidence regarding possible causes of the observed developments. However, it cannot be overlooked that 2020 to 2023 was a time characterised by multiple crises: the COVID-19 pandemic, wars which have had substantial impacts on the political climate and economy in Germany, worsening effects of the climate crisis, housing shortages, rising costs of living, inflation and recession in 2023 [71]. The association between economic downturn and declines in mental health has been repeatedly reported [72–74]. In addition, there are signs of a deterioration of social cohesion. In an online survey conducted in Germany in 2023, a large majority reported perceiving society as divided, feeling abandoned by politics and struggling to feel hopeful about the future [75]. Perceived egoism and social injustice increased substantially between 2020 and 2023 (ibid.). Analyses by the Federal Institute for Population Research based on data from three different population surveys showed a substantial rise in loneliness among adults aged 18–53 in 2020–2022 compared to 2005–2017 [76].

In addition to these changes in risk factors, the current discussion around the potential impacts of changing mental health literacy and awareness (perhaps influenced the COVID-19 pandemic [77]) as well as concomitant shifts of mental disorder concepts should be taken into account when considering possible explanations for findings of rising morbidity [78–81]. However, while there is evidence that self-diagnoses of mental disorders become more likely with broader concepts of disorders [81], it remains to be examined whether self-reports regarding specific symptoms are also subject to such effects. Potential changes in the willingness to disclose symptoms are also possible but underexamined.

Consistent with previous studies [9, 13, 14, 82], we find a clear and unchanged social gradient to the disadvantage of those with lower educational attainment. While our analyses do not show trend differences between education groups for 2020 to 2023, the development of social inequality in mental health should be watched closely in light of ongoing crises, a rise in the symptom of low self-worth or guilt in the low level of education group and a striking increase in symptoms of depression among males in the low level of education group in the most recent observation period. Males with low educational attainment have been found to be especially vulnerable to mental health declines in times of increased economic hardship previously [83]. We also find a striking increase in severe symptoms among males 2022 to 2023, but not among females. In general, the possibility of gender-specific changes in self-reports on depressive symptoms should be examined in light of evolving gender roles (see also discussion of reductions in the gender gap in depression diagnoses [30]). However, these increases seen only in the final year of the observation period against a backdrop of developments known to be risk factors for males with a low level of education in particular is more straightforwardly interpretable as evidence for a decline in mental health. Overall, we find the expected gender gap to the disadvantage of females [1], with young women particularly affected in the final observation period. With regard to comparisons between age groups, our results show that the youngest adults appear to be overall more at risk of depressive symptoms than the eldest adults, which was not the pattern found in the total European sample of the EHIS 2014/2015 [84] or 2019/2020 [85] but also reported in a large-scale German cohort study with data collected 2014–2019 [9].

The observed recent developments in Germany do not appear to have occurred across western countries. Data from Belgium shows fluctuations including peaks in pandemic times with no clear overall trajectory 2019 to 2022 [25], albeit a clear increase compared to pre-2019 [27]. Data from France [24] and the USA [26] shows generally lower symptom levels in 2022/2023 than in 2020/2021. However, symptom prevalence in Germany in 2022 to 2023 did not surpass prevalence in the United States or Belgium, for which PHQ-based estimates were reported: mostly between 20 and 25% positive PHQ-2 screens in the USA [26] and 13–16% positive PHQ-9 screens in Belgium in this time period [25] is at or above levels found in Germany (present study and PHQ-2 results in [46, 47, 67]), albeit using different survey methods.

## Limitations

Apart from the survey design changes between the pre-2019 surveys discussed above, limitations of the present study also include the underrepresentation of certain population subgroups in the surveys analysed, particularly of younger adults and those with lower levels of education. While weighting procedures were used to adjust the sample to the population structure, smaller sample sizes in these groups result in greater statistical uncertainty and make changes and differences more difficult to detect. The exclusion of institutionalised individuals from the survey samples and the possibility of selective non-response among those with compromised (mental) health [86, 87], both of which cannot be corrected for, may result in an underestimation of the prevalence of depressive symptoms. Furthermore, self-report can be subject to recall bias and influences of social desirability [88], which may also lead to underestimation. On the other hand, the PHQ-8 has been found to produce more false positives as a screening instrument for major depression in general population samples than in clinical samples [89], meaning that a certain degree of overestimation with regard to the clinical significance of the reported symptoms may also be at play.

## Conclusion

The observed increases in prevalence of symptoms of depression among adults in Germany 2008–2023 and particularly in the final years of the observation period characterised by multiple collective crises call for efforts to promote and protect the mental health of the population. Especially in light of the socially unequal distribution of symptoms of depression across all observation periods, health in all policies should be a guiding principle and interventions should address social determinants of mental health [90]. Continued systematic surveillance of symptoms of depression and other mental health indicators is needed and should aim to settle on one measurement methodology to be applied consistently over time for each indicator.

## Electronic supplementary material

Below is the link to the electronic supplementary material.

Supplementary Material 1

## Data Availability

Population-based data from the German health monitoring program that support the findings of this study are available from the Robert Koch Institute (RKI) but restrictions apply to the availability of these data, which were used under license for the current study and so are not publicly available. The data set cannot be made publicly available because informed consent from study participants did not cover public deposition of data. However, a minimal data set is archived in the Health Monitoring Research Data Centre at the RKI and can be accessed by all interested researchers. On-site access to the data set is possible at the Secure Data Centre of the RKI’s Health Monitoring Research Data Centre. Requests should be submitted to the Health Monitoring Research Data Centre, Robert Koch Institute, Berlin, Germany (Email: fdz@rki.de).
