# Supplementary material for: Trends in depressive symptoms in Germany’s adult population 2008–2023
Source: Soc Psychiatry Psychiatr Epidemiol. 2025 Jul 22;61(3):435–47. doi: 10.1007/s00127-025-02965-6 (PMC12995932; doi:10.1007/s00127-025-02965-6)
Supplement: Supplementary file 1 — Supplementary Material 1 [file 127_2025_2965_MOESM1_ESM.docx]

Supplementary Material: Trends in depressive symptoms in the German adult population 2008-2023

Lena Walther^1*^, Stephan Junker^1^, Petra Rattay^1^, Ronny Kuhnert^1^, Heike Hölling^1^, Elvira Mauz^1^

1 Department of Epidemiology and Health Monitoring, Robert Koch-Institute, Berlin, Germany

**Correspondence:* Lena Walther*,* [WaltherL@rki.de](mailto:WaltherL@rki.de)

**Table 1: Unstandardised, weighted percentages of PHQ-8 ≥ 10 and PHQ-8 ≥ 15 by survey**

| **survey** | **percent PHQ-8 ≥ 10** | **lower 95% CI limit** | **upper 95% CI limit** | **percent PHQ-8 ≥ 15** | **lower 95% CI limit** | **upper 95% CI limit** |
| --- | --- | --- | --- | --- | --- | --- |
| 2008-2011 | 7.4% | 6.5% | 8.3% | 1.6% | 1.2% | 2.1% |
| 2014-2015 | 9.9% | 9.3% | 10.4% | 3.2% | 2.9% | 3.5% |
| 2019-2020  (a) | 9.1% | 8.3% | 10.1% | 3.1% | 2.6% | 3.7% |
| 2020 (b) | 7.6% | 6.7% | 8.5% | 2.6% | 2.1% | 3.2% |
| 2021 | 10.5% | 9.0% | 12.2% | 3.2% | 2.4% | 4.2% |
| 2022 | 13.4% | 12.1% | 14.8% | 3.7% | 3.0% | 4.5% |
| 2023 | 14.7% | 13.5% | 16.0% | 4.7% | 4.0% | 5.5% |

**Table 2: Results of logistic regressions with linear splines for two time periods (2008-2020; 2020-2023) predicting PHQ-8 ≥ 10**

| **model** | **term** | **estimate** | **standard error** | **p-value** | **p-value slope 2 versus slope 1** |
| --- | --- | --- | --- | --- | --- |
| total | slope 1 | -0.002 | 0.007 | 0.784 |  |
|  | slope 2 | 0.206 | 0.022 | <0.001 | <0.001 |
| by sex | slope 1 (m) | 0.010 | 0.010 | 0.318 |  |
|  | slope 2 (m) | 0.201 | 0.034 | <0.001 | <0.001 |
|  | slope 1 (f vs. m) | -0.022 | 0.013 | 0.088 |  |
|  | slope 2 (f vs. m) | 0.009 | 0.044 | 0.838 | 0.557 |

Weighted, age- and sex-standardised data. Slope 1 represents linear spline for 2008-2011 survey to 2020 b) survey; slope 2 represents linear spline for 2020 b) survey to 2023 survey. Only results for linear spline terms shown.

**Table 3: Trends PHQ-8 = 10-14**

| **model** | **term** | **estimate** | **standard error** | **p-value** | **p-value slope 2 versus slope 1** |
| --- | --- | --- | --- | --- | --- |
| total | slope 1 | -0.012 | 0.008 | 0.124 |  |
|  | slope 2 | 0.221 | 0.025 | <0.001 | <0.001 |
| by sex | slope 1 (m) | 0.006 | 0.012 | 0.605 |  |
|  | slope 2 (m) | 0.183 | 0.039 | <0.001 | <0.001 |
|  | slope 1 (f vs. m) | -0.033 | 0.015 | 0.030 |  |
|  | slope 2 (f vs. m) | 0.069 | 0.050 | 0.172 | 0.092 |

Weighted, age- and sex-standardised data. Slope 1 represents linear spline for 2008-2011 survey to 2020 b) survey; slope 2 represents linear spline for 2020 b) survey to 2023 survey. Only results for linear spline terms shown.

**Table 4: Trends PHQ-8 ≥ 15**

| **model** | **term** | **estimate** | **standard error** | **p-value** | **p-value slope 2 versus slope 1** |
| --- | --- | --- | --- | --- | --- |
| total | slope 1 | 0.021 | 0.012 | 0.082 |  |
|  | slope 2 | 0.140 | 0.040 | <0.001 | 0.011 |
| by sex | slope 1 (m) | 0.018 | 0.017 | 0.290 |  |
|  | slope 2 (m) | 0.208 | 0.062 | 0.001 | 0.009 |
|  | slope 1 (f vs. m) | 0.005 | 0.023 | 0.833 |  |
|  | slope 2 (f vs. m) | -0.125 | 0.080 | 0.116 | 0.169 |

Weighted, age- and sex-standardised data. Slope 1 represents linear spline for 2008-2011 survey to 2020 b) survey; slope 2 represents linear spline for 2020 b) survey to 2023 survey. Only results for linear spline terms shown.

**Table 5: Trends PHQ-8 ≥ 15 compared to PHQ-8 = 10-14**

| **model** | **term** | **estimate** | **standard error** | **p-value** | **p-value slope 2 versus slope 1** |
| --- | --- | --- | --- | --- | --- |
| total | slope 1 | 0.035 | 0.015 | 0.021 |  |
|  | slope 2 | -0.074 | 0.048 | 0.122 | 0.062 |
| by sex | slope 1 (m) | 0.013 | 0.023 | 0.573 |  |
|  | slope 2 (m) | 0.028 | 0.074 | 0.702 | 0.866 |
|  | slope 1 (f vs. m) | 0.039 | 0.031 | 0.201 |  |
|  | slope 2 (f vs. m) | -0.190 | 0.096 | 0.049 | 0.052 |

Weighted, age- and sex-standardised data. Slope 1 represents linear spline for 2008-2011 survey to 2020 b) survey; slope 2 represents linear spline for 2020 b) survey to 2023 survey. Only results for linear spline terms shown.

**Table 6: Trends PHQ-8 ≥ 10 by age and by age and sex**

| **model** | **term** | **estimate** | **standard error** | **p-value** | **p-value slope 2 versus slope 1** |
| --- | --- | --- | --- | --- | --- |
| by age | slope 1 (18-29 y) | -0.011 | 0.015 | 0.441 |  |
|  | slope 2 (18-29 y) | 0.254 | 0.052 | <0.001 | <0.001 |
|  | slope 1 (30-44y vs. 18-29 y) | <0.001 | 0.020 | 0.982 |  |
|  | slope 2 (30-44y vs. 18-29 y) | -0.012 | 0.068 | 0.864 | 0.882 |
|  | slope 1 (45-64 y vs. 18-29 y) | 0.022 | 0.017 | 0.207 |  |
|  | slope 2 (45-64 y vs. 18-29 y) | -0.104 | 0.061 | 0.088 | 0.083 |
|  | slope 1 (65-79 y vs. 18-29 y) | 0.014 | 0.023 | 0.528 |  |
|  | slope 2 (65-79 y vs. 18-29 y) | -0.055 | 0.071 | 0.440 | 0.426 |
| females by age | slope 1 (18-29 y) | -0.007 | 0.019 | 0.695 |  |
|  | slope 2 (18-29 y) | 0.246 | 0.069 | <0.001 | 0.002 |
|  | slope 1 (30-44y vs. 18-29 y) | -0.011 | 0.025 | 0.680 |  |
|  | slope 2 (30-44y vs. 18-29 y) | -0.016 | 0.087 | 0.855 | 0.959 |
|  | slope 1 (45-64 y vs. 18-29 y) | 0.004 | 0.022 | 0.864 |  |
|  | slope 2 (45-64 y vs. 18-29 y) | -0.086 | 0.080 | 0.280 | 0.340 |
|  | slope 1 (65-79 y vs. 18-29 y) | -0.018 | 0.028 | 0.514 |  |
|  | slope 2 (65-79 y vs. 18-29 y) | 0.009 | 0.090 | 0.923 | 0.802 |
| males by age | slope 1 (18-29 y) | -0.018 | 0.024 | 0.454 |  |
|  | slope 2 (18-29 y) | 0.275 | 0.080 | 0.001 | 0.002 |
|  | slope 1 (30-44y vs. 18-29 y) | 0.015 | 0.032 | 0.632 |  |
|  | slope 2 (30-44y vs. 18-29 y) | -0.016 | 0.106 | 0.876 | 0.801 |
|  | slope 1 (45-64 y vs. 18-29 y) | 0.044 | 0.027 | 0.105 |  |
|  | slope 2 (45-64 y vs. 18-29 y) | -0.134 | 0.094 | 0.154 | 0.111 |
|  | slope 1 (65-79 y vs. 18-29 y) | 0.053 | 0.037 | 0.144 |  |
|  | slope 2 (65-79 y vs. 18-29 y) | -0.134 | 0.113 | 0.233 | 0.175 |

Weighted, age- and sex-standardised data. Slope 1 represents linear spline for 2008-2011 survey to 2020 b) survey; slope 2 represents linear spline for 2020 b) survey to 2023 survey. Only results for linear spline terms shown.

**Table 7: Trends PHQ-8 ≥ 10 by education and by education and sex**

| **model** | **term** | | **estimate** | **standard error** | **p-value** | **p-value slope 2 versus slope 1** |
| --- | --- | --- | --- | --- | --- | --- |
| by education group | slope 1 (low) | | 0.017 | 0.012 | 0.162 |  |
|  | slope 2 (low) | | 0.238 | 0.043 | <0.001 | <0.001 |
|  | slope 1 (medium vs. low) | | -0.019 | 0.015 | 0.188 |  |
|  | slope 2 (medium vs. low) | | -0.028 | 0.052 | 0.585 | 0.885 |
|  | slope 1 (high vs. low) | | -0.028 | 0.018 | 0.113 |  |
|  | slope 2 (high vs. low) | | -0.031 | 0.056 | 0.581 | 0.965 |
| females by education | slope 1 (low) | 0.005 | | 0.016 | 0.748 |  |
|  | slope 2 (low) | 0.175 | | 0.061 | 0.004 | 0.017 |
|  | slope 1 (medium vs. low) | -0.018 | | 0.020 | 0.370 |  |
|  | slope 2 (medium vs. low) | 0.059 | | 0.071 | 0.406 | 0.360 |
|  | slope 1 (high vs. low) | -0.007 | | 0.024 | 0.760 |  |
|  | slope 2(high vs. low) | 0.064 | | 0.077 | 0.411 | 0.439 |
| males by education | slope 1 (low) | 0.032 | | 0.017 | 0.064 |  |
|  | slope 2 (low) | 0.287 | | 0.060 | <0.001 | <0.001 |
|  | slope 1 (medium vs. low) | -0.021 | | 0.022 | 0.341 |  |
|  | slope 2 (medium vs. low) | -0.108 | | 0.076 | 0.153 | 0.334 |
|  | slope 1 (high vs. low) | -0.058 | | 0.025 | 0.022 |  |
|  | slope 2 (high vs. low) | -0.140 | | 0.079 | 0.079 | 0.389 |

Weighted, age- and sex-standardised data. Slope 1 represents linear spline for 2008-2011 survey to 2020 b) survey; slope 2 represents linear spline for 2020 b) survey to 2023 survey. Only results for linear spline terms shown.

**
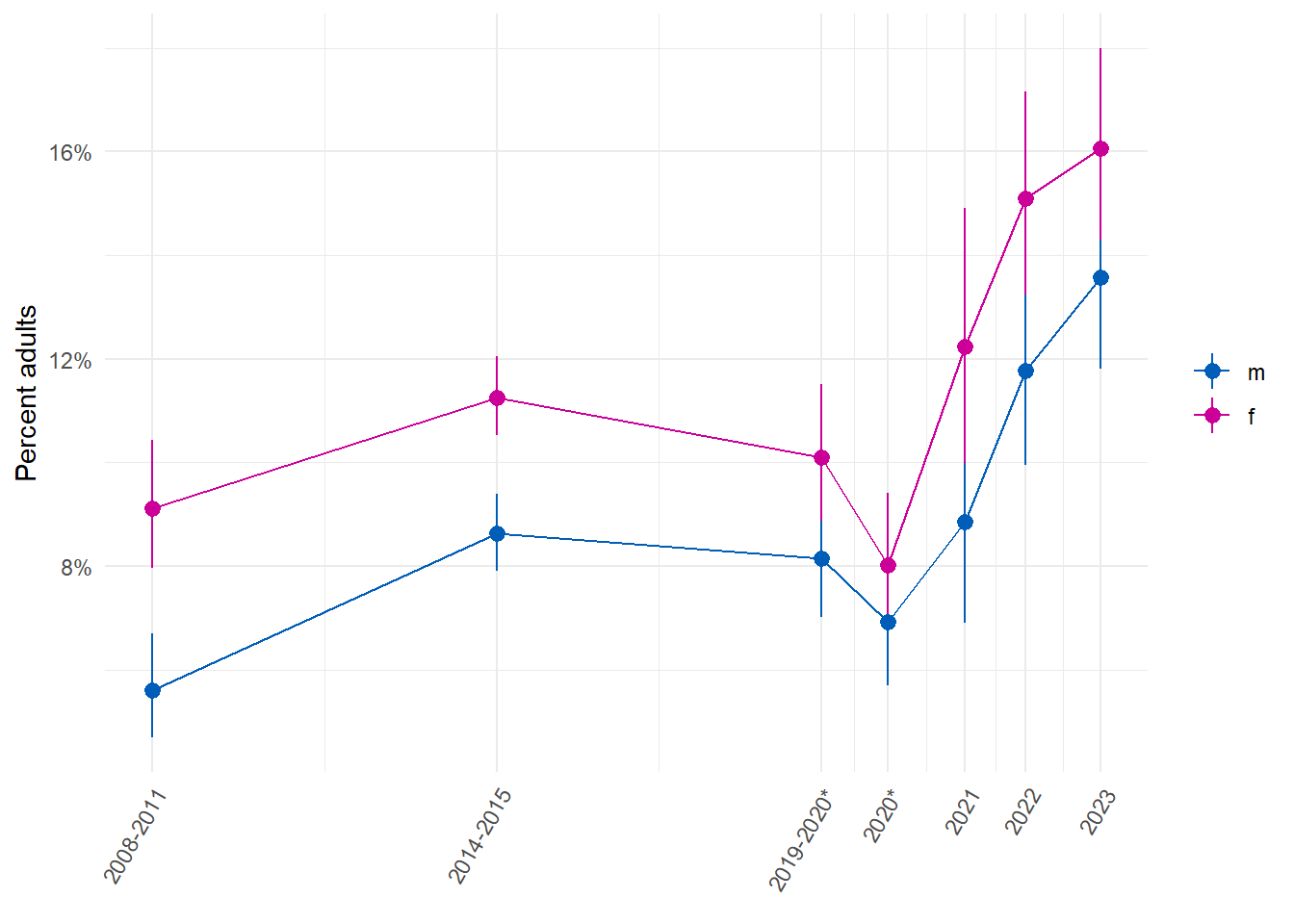
Figure 1: Depressive symptoms stratified by sex 2008-2023**

Weighted and age-standardised percentage of females and males with symptoms of depression (PHQ-8 ≥ 10). For the purposes of representing time between surveys, we plotted each estimate at the temporal medium of the respective survey’s field time. *2019-2020 covers a pre-COVID-19 pandemic period (4/19-3/20), *2020 covers a peri-pandemic period (3/20-1/21). There are survey design differences between the 2008-2011, the 2014-2015 survey, and the 2019-2023 surveys (constant methodology 2019-2023) (see Methods).

**Figure 2: Individual depressive symptoms stratified by age 2008-2023**

Weighted and sex-standardised percentage of adults aged 18-29 (**A**), 30-44 (**B**), 45-64 (**C**), 65-79 (**D**) reporting a frequency of “more than half the days” or “nearly every day” in the previous two weeks for each of the eight symptoms captured by the PHQ-8. *2019-2020 covers a pre-COVID-19 pandemic period (4/19-3/20), *2020 covers a peri-pandemic period (3/20-1/21). There are survey design differences between the 2008-2011, the 2014-2015 survey, and the 2019-2023 surveys (constant methodology 2019-2023) (see Methods).

**a 18-29 years**

**
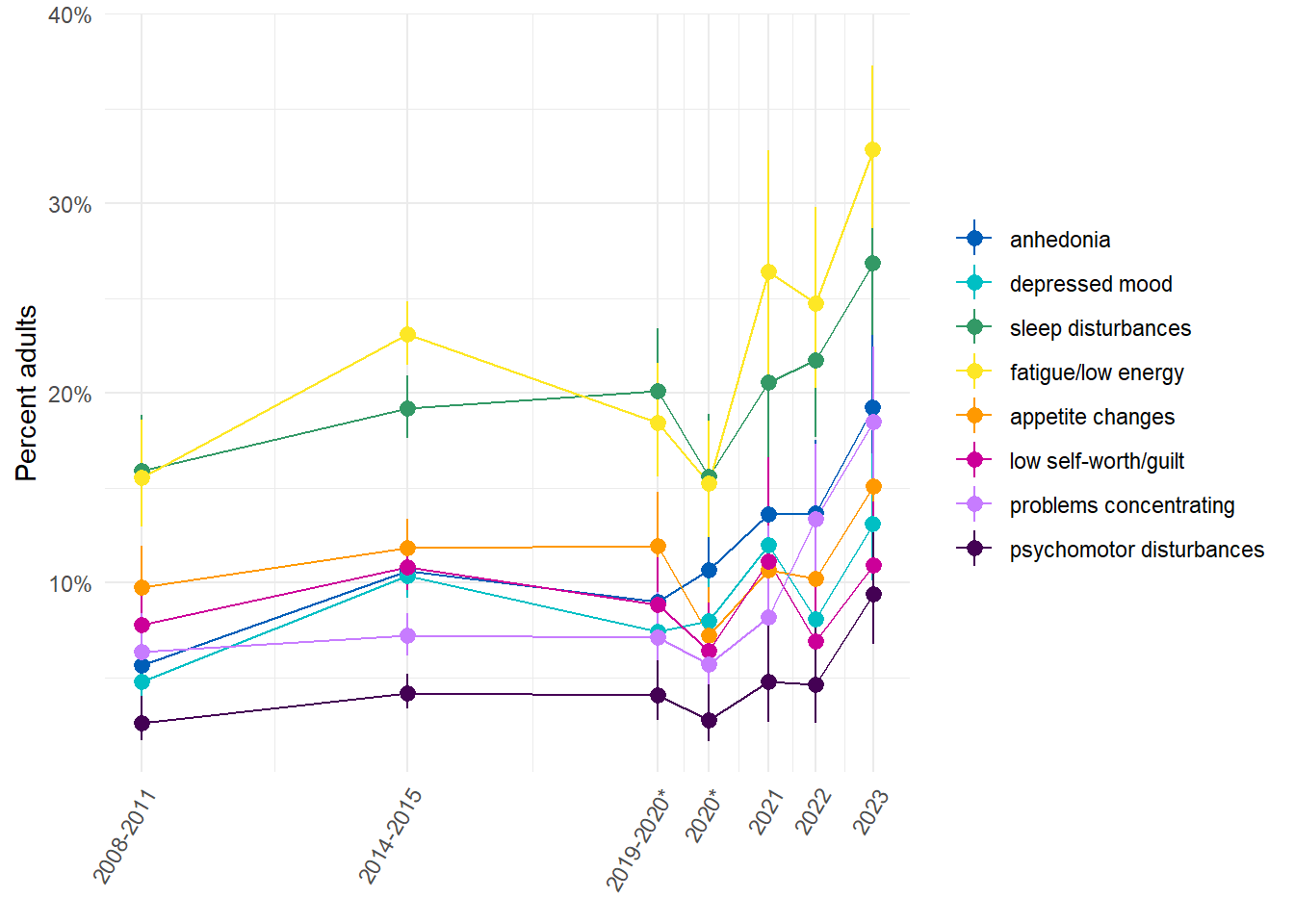
**

**b 30-44 years**

**
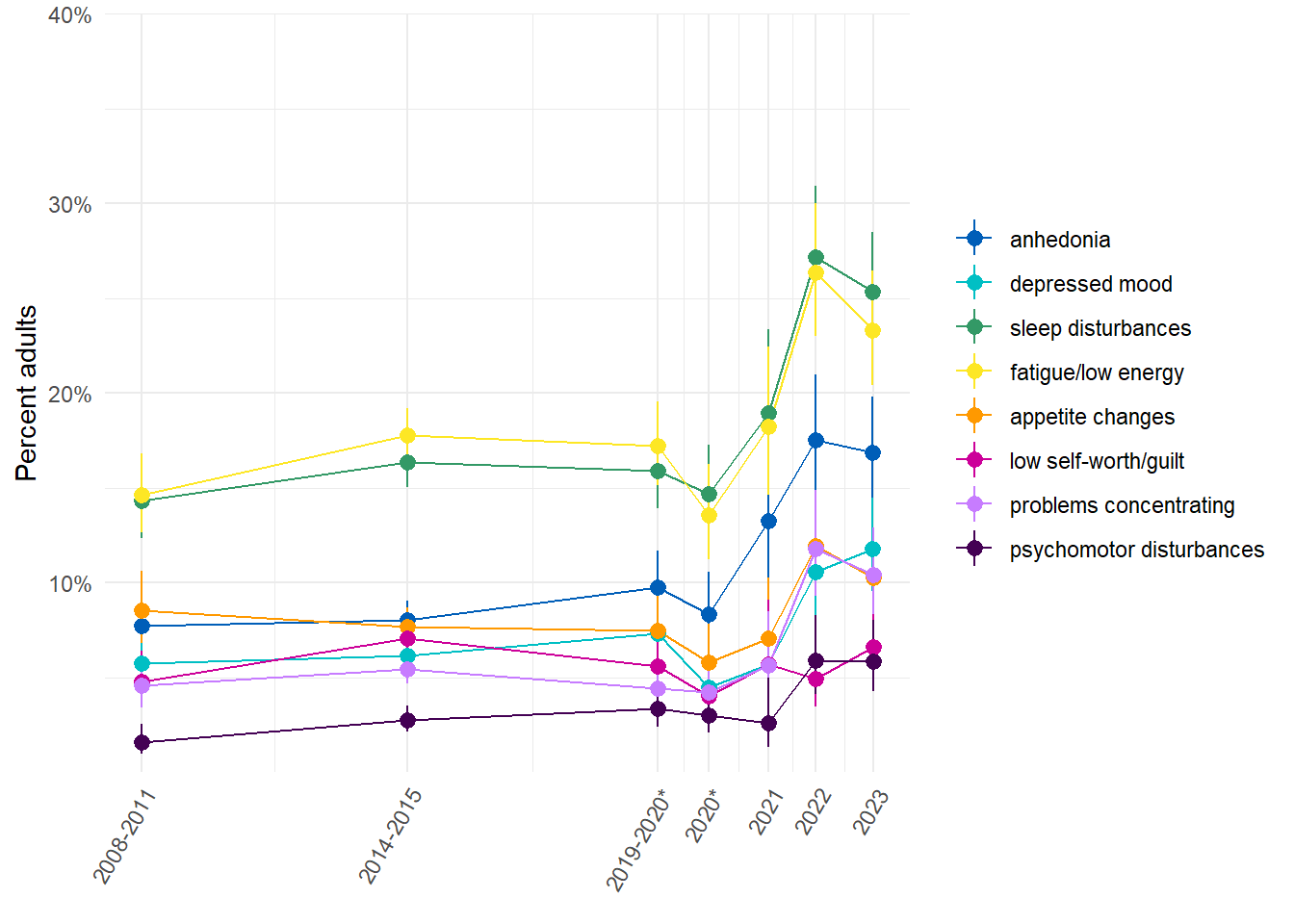
**

**c 45-64 years**

**
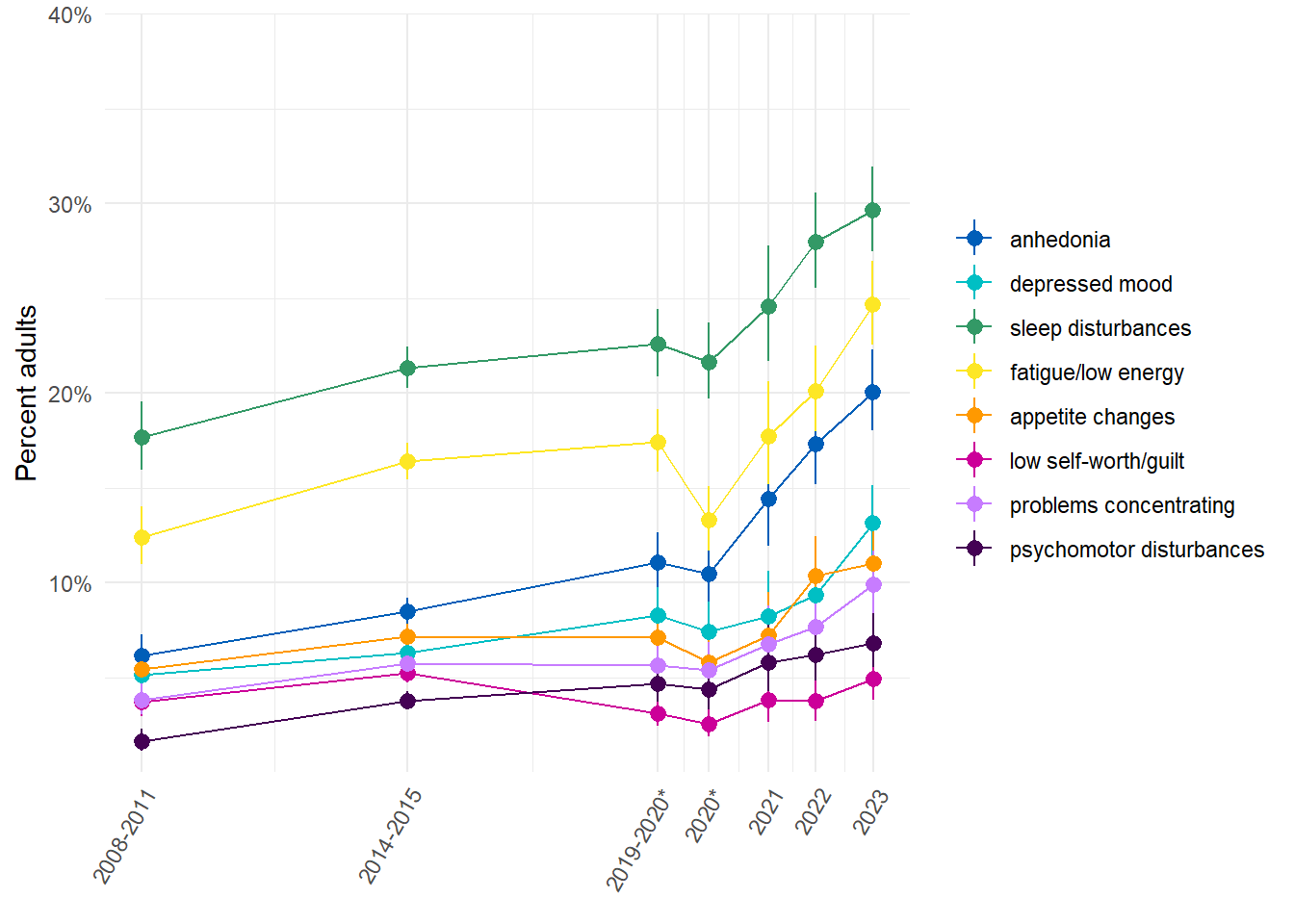
**

**d 65-79 years**

**
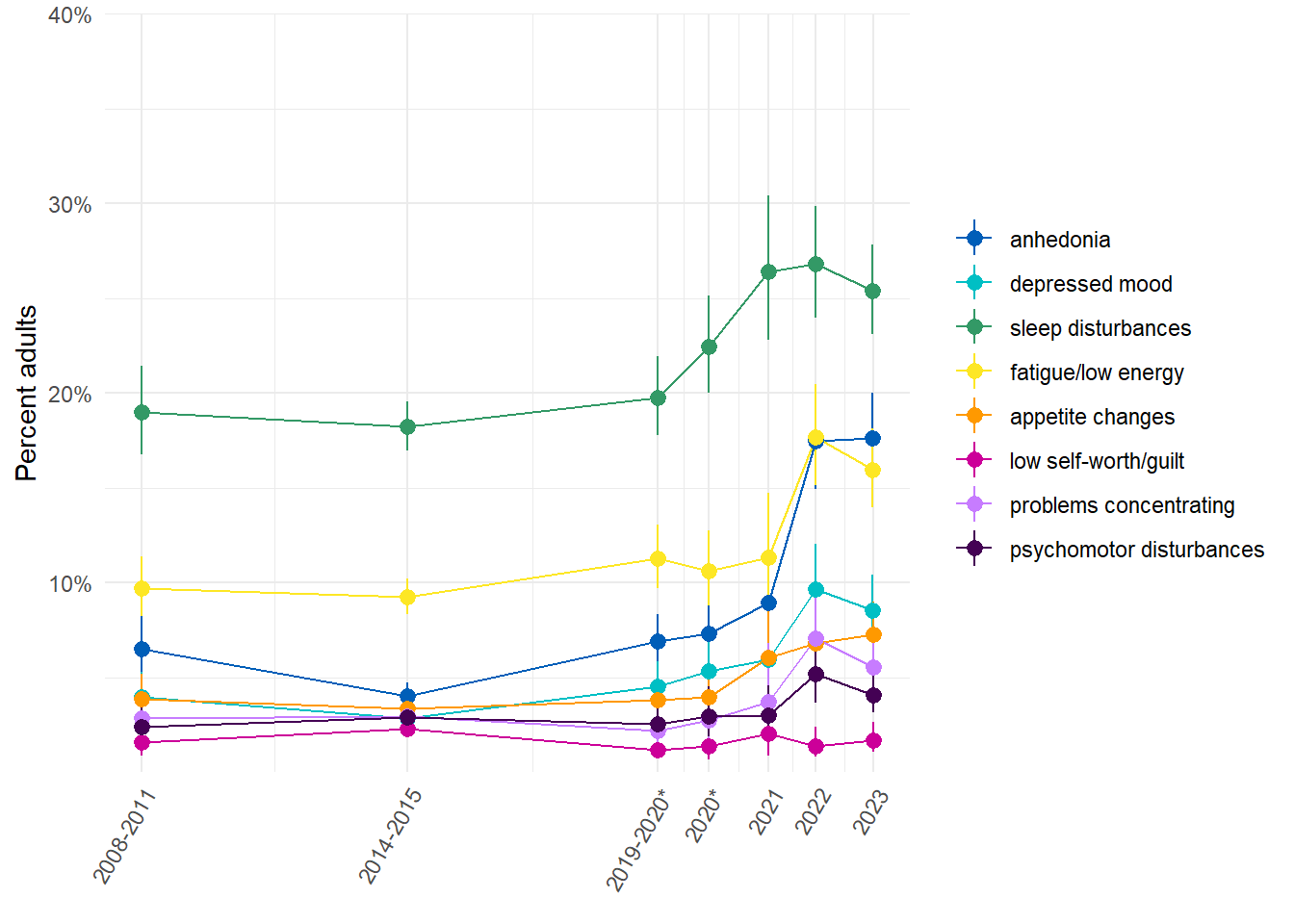
**

**Figure 3: Individual depressive symptoms stratified by education 2008-2023**

Weighted and sex- and age-standardised percentage of adults with low (**A**), medium (**B**), high (**C**) level of education reporting a frequency of “more than half the days” or “nearly every day” in the previous two weeks for each of the eight symptoms captured by the PHQ-8. *2019-2020 covers a pre-COVID-19 pandemic period (4/19-3/20), *2020 covers a peri-pandemic period (3/20-1/21). There are survey design differences between the 2008-2011, the 2014-2015 survey, and the 2019-2023 surveys (constant methodology 2019-2023) (see Methods).

**a Low level of education**

**
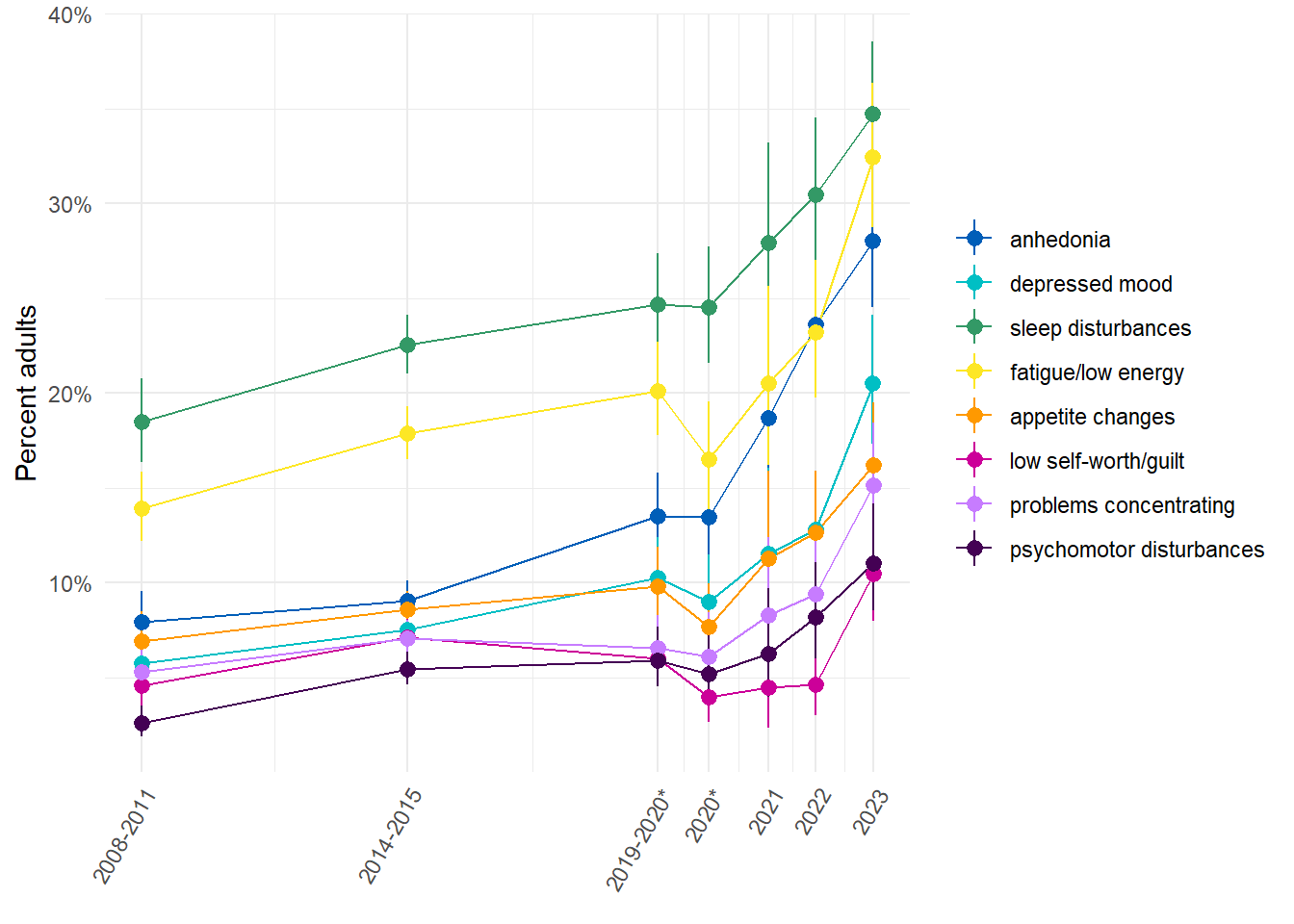
**

**b Medium level of education**

**
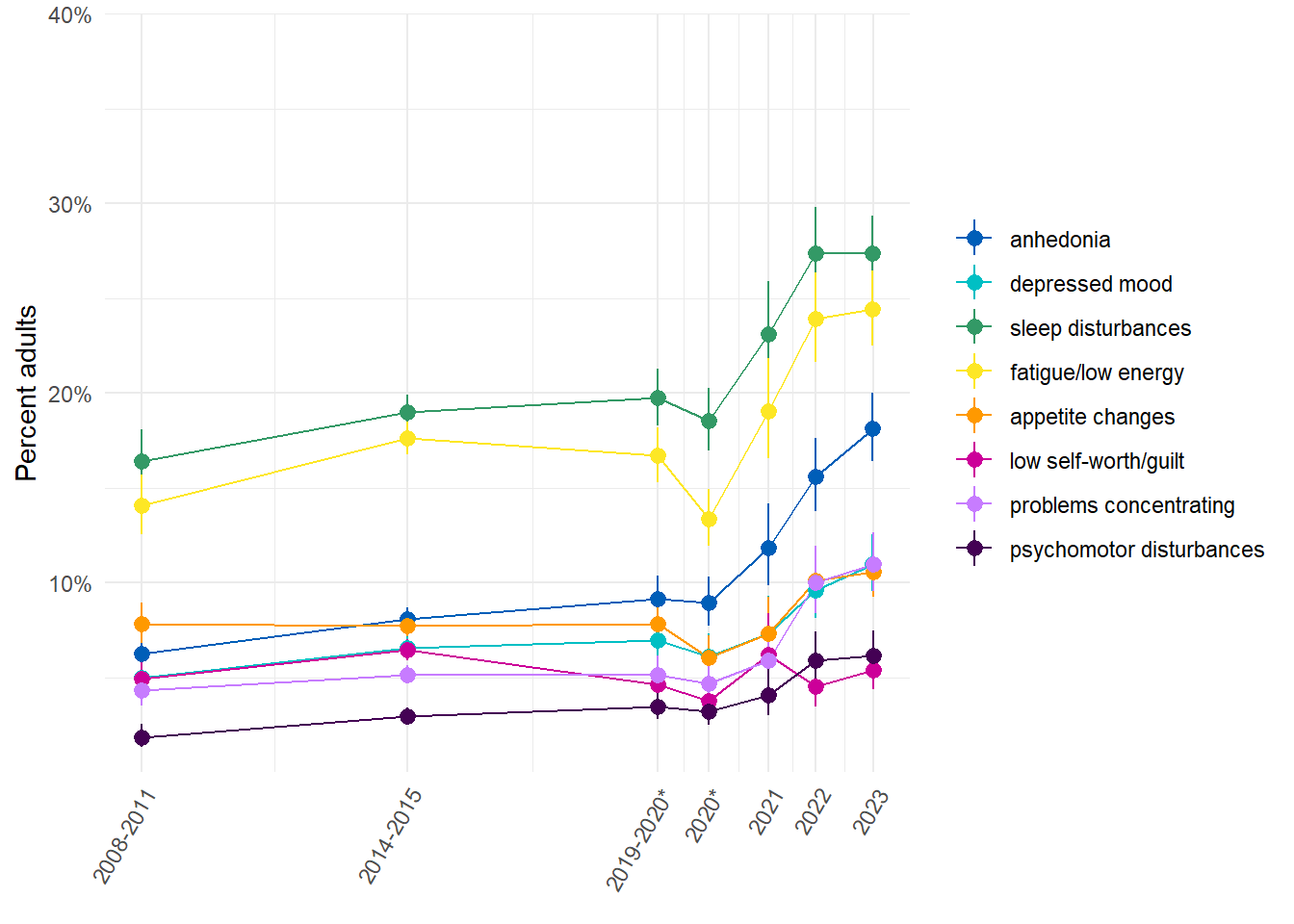
**

**c High level of education**

**
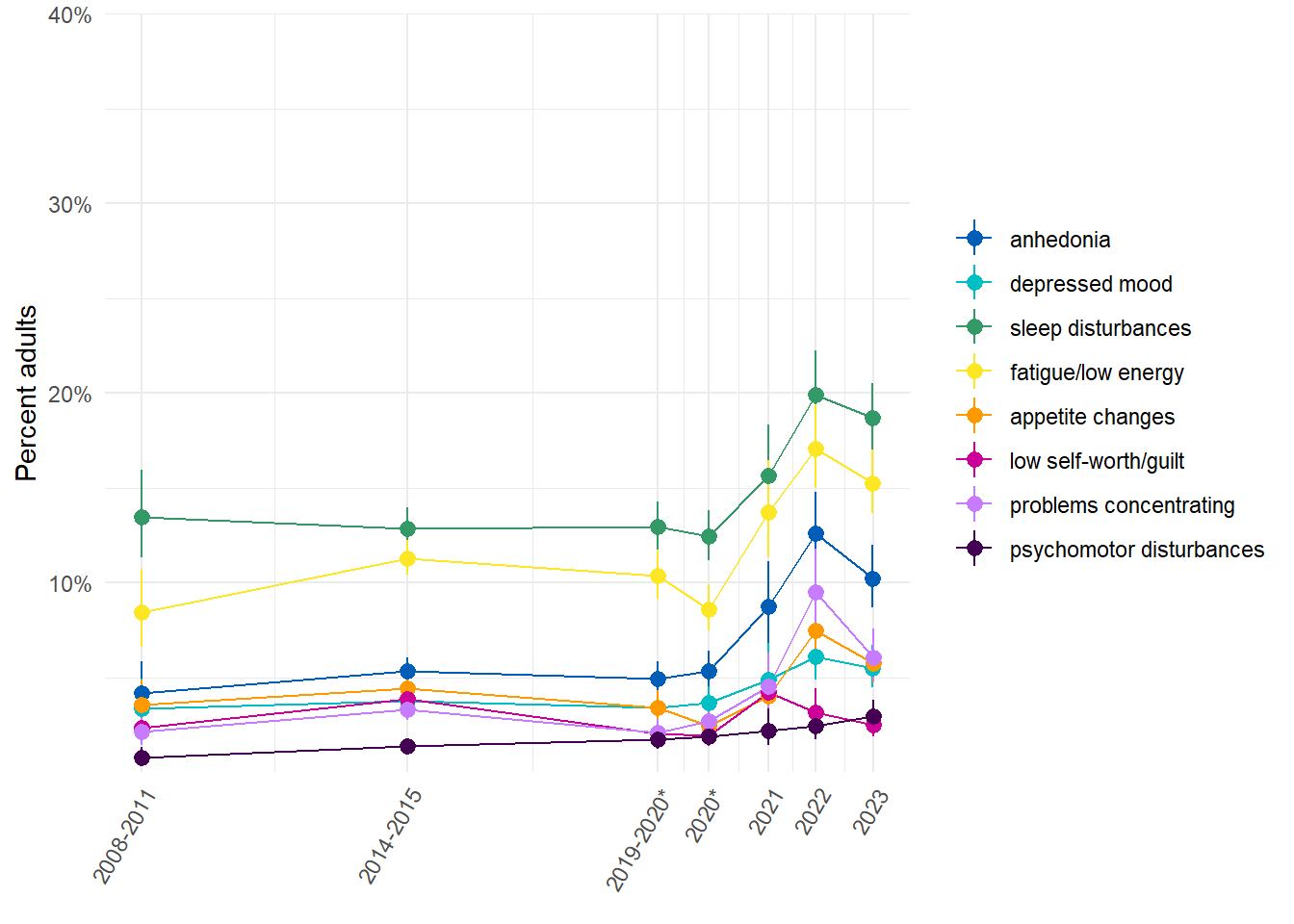
**
